# An Extensive Study Regarding the Microscopic Anatomy of the Early Fetal Human Optic Nerve

**DOI:** 10.3390/neurolint16030035

**Published:** 2024-04-24

**Authors:** Mihai Alin Publik, Florin Mihail Filipoiu, Adrian Vasile Dumitru, Andrei Precup, Ioan-Andrei Petrescu, Iulian Slavu, Raluca Florentina Tulin, Adrian Tulin, Andra Ioana Baloiu, Monica Mihaela Cirstoiu, Octavian Munteanu

**Affiliations:** 1 Department of Anatomy, “Carol Davila” University of Medicine and Pharmacy, 050474 Bucharest, Romania; mihai.publik@stud.umfcd.ro (M.A.P.); florin.filipoiu@umfcd.ro (F.M.F.); andrei.precup@stud.umfcd.ro (A.P.); iulian.slavu@umfcd.ro (I.S.); raluca_2lin@yahoo.com (R.F.T.); adrian.tulin@umfcd.ro (A.T.); baloiu.andra@gmail.com (A.I.B.); octav_munteanu@yahoo.com (O.M.); 2 Department of Pathology, “Carol Davila” University of Medicine and Pharmacy, 050474 Bucharest, Romania; dr.adriandumitru@yahoo.com; 3 Department of Obstetrics and Gynecology, “Carol Davila” University of Medicine and Pharmacy, 050474 Bucharest, Romania; dr_cirstoiumonica@yahoo.com

**Keywords:** optic nerve, development, blood–nerve barrier, glia limitans, embryology, S100 protein, astrocyte, GFAP, E-cadherin, eye, meningeal primordium

## Abstract

The development of the optic nerve and its surrounding tissues during the early fetal period is a convoluted period because it spans both the organogenesis period and the fetal period. This study details the microscopic anatomy and histoembryology of the optic nerve in embryos during the early fetal period, including the second half of the first trimester of pregnancy. Serial sections through the orbit of variously aged embryos allowed us to analyze the nerve in both longitudinal and transverse aspects. A histological assessment and description of the structures surrounding and inside the nerve were performed, highlighting the cellular subtypes involved. By employing immunohistochemical techniques, we could characterize the presence and distribution of astrocytes within the optic nerve. Our findings suggest that by the 8th gestational week (WG) the structures are homologs to all the adult ones but with an early appearance so that maturation processes take place afterward. By this age, the axons forming the nerve are definitive adult axons. The glial cells do not yet exhibit adult phenotype, but their aspect becomes adult toward the 13th week. During its development the optic nerve increases in size then, at 14 weeks, it shrinks considerably, possibly through its neural maturation process. The morphological primordium of the blood–nerve barrier can be first noted at 10 WG and at 13 WG the morphological blood–nerve barrier is definitive. The meningeal primordium can be first noted as a layer of agglomerated fibroblasts, later toward 13 WG splitting in pachymeninx and leptomeninges and leaving space for intrinsic blood vessels.

## 1. Introduction

The embryology of the optic nerve (ON) is an extensive field of research due to its intricate histogenesis and histoembryology in the first place and, moreover, the complex physiological function in postpartum life. From the very beginning of its formation and until its complete development, the ON is one outstanding structure with an embryology, function, and histology linked to the central nervous system.

**Figure 1:**
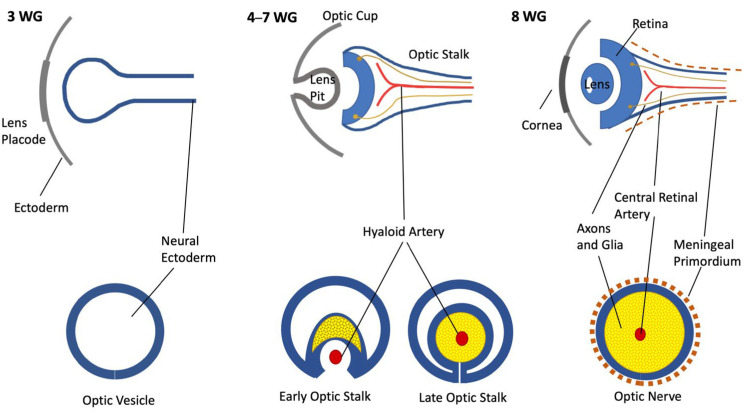
Schematic representation of ON development. Upper row: coronal section; bottom row: ON section of each gestational age.

The development of the nervous structures of the eye, namely, the ON and the retina, is the result of the synchronous development of the forebrain beginning in the early 3rd gestational week. The forebrain forms two evaginations called optic vesicles that by the end of the 4th week reach the ectoderm, forming the optic cups, bearing a link to the brain through the optic stalk (OS) [1,2]. The OS is hollow and has a cleft in the inferior part—the choroid fissure—where by the 7th week an artery and a vein will be enclosed, becoming the central retinal artery (CRA) and vein [3] (Figure 1).

Inside the mammalian nervous system, the first cells to be produced are the retinal ganglion cells, beginning by day 11, followed by the astrocytes and oligodendrocytes [4,5,6]. For the ON, the neuroglia arise from the walls of the OS in the form of glioblasts, which will rapidly begin to undergo division radially [1,4,7]. During this period, an increasing number of axons from the ganglion cells invade the OS to form the ON, navigating toward the future optic chiasm [8]. An overproduction of axons occurs, and many of them immediately enter apoptosis [9]. This process is definitive by the 29th week of gestation when approximately 70% of the formed axons will degenerate, leaving the fetus with the adult number of axons in the ON, which is circa 1.1 million [9]. This represents a fine-tuning process through which the axons redirect toward specific loci in the lateral geniculate nucleus, reshaping the retinal map [9,10,11].

The surrounding mesenchyme (i.e., mesoderm), along with cells of neuroepithelial origin, forms the meninges through a condensation process that will later separate in all three layers [1,12,13]. The nerve exits the eye through the optic disk and traverses the layers of the eye through lamina cribrosa, then going through the orbit toward the posterior and lateral aspects of the structure [10,14]. Numerous blood vessels nourish the retina, but only in the 4th month of gestation do capillaries branch out and grow inside the retina [15,16,17].

The development of the ON has been a matter of concern to the extent that the cellular aspect in ON embryology has been studied both in in vivo and in vitro environments and the intricate molecular factors governing the process have been vigorously brought to light. Yet, comprehensive approaches in the literature are rather scarce. This study aims to expound the morphogenesis of the early fetal optic nerve by means of the morphological, embryological, and ultrastructural changes that take place during this period.

## 2. Materials and Methods

The current study was performed at the Anatomy and Embryology Department of “Carol Davila” University of Medicine and Pharmacy Bucharest. Between 1 January 2017 and 29 August 2021, 30 women admitted to the Department of Obstetrics and Gynecology of University Emergency Hospital in Bucharest, with a singleton pregnancy, who had a spontaneous abortion, and donated the embryos for this study under personal consent and according to the current Romanian and European regulations and bioethics requirements. The 30 aborted embryos had a gestational age varying between 8 and 14 WG and no structural external or internal abnormalities. The age of each specimen was established by crown–rump length measurements according to the Carnegie staging system and included in a group based on age (+/− one week) resulting in the following: 8 WG (9 specimens), 10 WG (10 specimens), and 13–14 WG (11 specimens divided into 6–13 WG and the other 5 being 14 WG) groups.

The specimens were fixed in 10% neutral buffered formalin for more than 1 year. The orbits were isolated by sectioning through the median plane and a horizontal plane below the maxillary bone and mounted for frontal and transversal histological slides. Therefore, 60 orbits including nerves, pertaining to the 30 embryos, were included in the study and analyzed in both frontal and transversal views. The pieces were processed classically for paraffin embedding then sectioned at either a 3- or 5-micron thickness each and were stained with the hematoxylin–eosin (HE) technique. Every slide was deparaffinized by incubation at 60 °C. All the slides were deparaffinized through two successive xylene baths. The rehydration was realized by three ethanol baths (100%, 96%, and 70%) and, finally, water wash. The slides were incubated in Meyer hematoxylin solution for 3 min, water washed, and then differentiated in mild hydrochloric acid in ethanol solution for 1 min and water washed again. Bluing was achieved by immersing the slides in tap water for 10 min. After a brief water wash, eosin solution was used next for 5 min. A wash step was performed; then, the final dehydration steps were carried out in successive ethanol solutions (70%, 95%, and 100%). Clarification was performed in two xylene baths; then, Entellan (Merck Millipore, Maharashtra, India, 107960) mounting medium and coverslip were lastly added.

The morphometric analysis of the ON size was carried out by choosing the equatorial slide for each transversally mounted orbit. We identified the section that had the CRA in the middle and also had the widest diameter of the ON. Once the equatorial slide was identified, we measured for every specimen the diameter of the ON exactly where it pierces the retinal pigmented epithelium. Then, a diameter was recorded for every orbit, calculating the average value for each age group. Also, the thickness of the retinal ganglion cell axon layer of the retina was measured at approximately 45° from the longitudinal axis of the ON.

A part of the sections was processed using BenchMark ULTRA IHC/ISH System (Roche Tissue Diagnostics, Almere, the Netherland) for obtaining immunohistochemistry-stained slides treated with the following primary HRP-conjugated antibodies: anti S100 calcium-binding protein (S100) (Ventana, Westmoreland County, PA, USA, 790-2914), neuron specific enolase (NSE) (Cell Marque, Rocklin, CA, USA, 760-4786), Glial Fibrillary Acidic Protein (GFAP) (Cell Marque, USA, 760-4345), and E-cadherin (Ventana, USA, 790-4497). All primary antibodies were prediluted and incubated using the automated stainer according to the producer. The detection system used was ultraView DAB Universal Detection System (Ventana, USA, 760-500). Appropriate positive controls (brain tissue from the same specimens) were also stained and assessed. The slides were evaluated with bright field microscopy, and micrographs of the representative fields were taken. Image acquisition was carried out using a Leica DMi8 inverted microscope in bright field mode. For the high-magnitude visualization (100× objective), we used Leica Microsystems Immersion Oil for Microscopes (Leica Microsystems, Wetzlar, Germany, 11513859). Images were processed in LAS X 3.0.13 software to obtain Z-Stack Images with processed Extended Depth of Field Images and also tile scan images, merged with the Mosaic Merge function, for overview micrographs.

## 3. Results

For a more didactic approach, our findings are presented in an antichronological order, as follows: firstly, 14 WG and 13 WG (due to the similarities with adult anatomy) and then 10 WG and 8 WG.

### 3.1. 14 WG and 13 WG Groups

#### 3.1.1. Surrounding Tissue and Elements

The vessels, nerves, and muscles have definitive positions, but, microscopically, we identified age-specific blastic attributes in the histology of the structures. The ON spans a transversal median value of 643.5 μm (IQR 8.5; *n* = 6) in the 13 WG specimens, whereas in the 14 WG, it measures a median value of 449 μm (IQR 7; *n* = 5).

**Figure 2:**
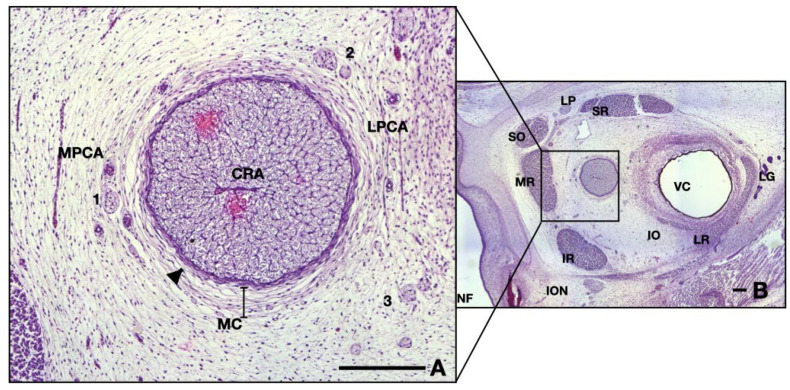
Frontal section of a 13 WG embryo orbit, HE staining: (**A**) detail of the ON and surrounding structures; (**B**) overview of the orbital space. Note the medial position of the nerve in the posterior half. CRA, central retinal artery; LPCA, lateral posterior ciliary artery; MPCA, medial posterior ciliary artery; MC, mesenchymal condensation; 1, nasociliary nerve; 2, long ciliary nerve; 3, branches of oculomotor nerve; arrowhead, epineurium; NF, nasal fossa; SR, superior rectus; LP, levator palpebrae; SO, superior oblique; MR, medial rectus; IR, inferior rectus; ION, infraorbital nerve; IO, inferior oblique; LR, lateral rectus; LG, lacrimal gland; VC, vitreous cavity. Scale bars: 250 μm.

Neighboring blood vessels respect the adult anatomy at this age, having a parallel pathway to the ON, lodged in the surrounding mesenchyme. Arteries can be divided into two different groups, as follows (see Figure 2A): on the right-hand side one can observe branches of lateral posterior ciliary artery, whereas on the left side are branches originating in the medial posterior ciliary artery. Note that the hyaloid vessels inside the vitreous are not present at this age (Figure 2B).

Small nervous branches are also visible in the neighboring region of the ON, also going parallel, along the nerve (see Figure 2A). Some nerves tend to pass through the mesenchyme along the arteries described anteriorly. The ones that we could identify were as follows: long ciliary nerves, medial to the ON; short ciliary nerves superior and laterally; and, also, branches of oculomotor nerve, inferior and laterally. Other very small branches can be seen in the micrograph, but their identification could not be achieved. They are probably ramifications of the long or short ciliary nerves.

**Figure 3:**
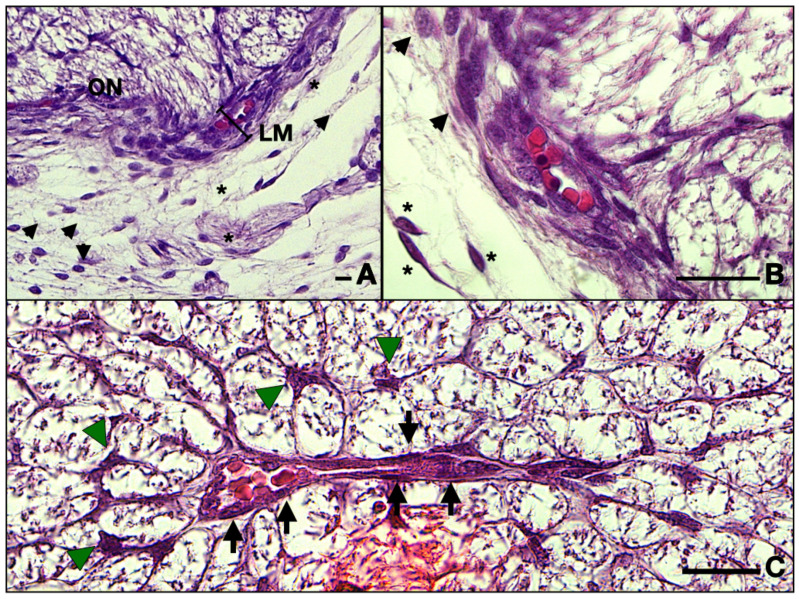
Frontal section of a 13 WG embryo orbit: (**A**) detail of the meninges and the leptomeningeal vessels; (**B**) detail of (**A**), and note the intimate relationships of the endothelial cells and fibroblasts; (**C**) micrographs of the central retinal artery inside the nerve. Note how the astrocyte bodies extend foot processes toward the artery radially. The endothelial cells border the lumen of the vessel. ON, optic nerve; LM, leptomeninges; black arrowhead, fibroblasts; asterisk, fibrocytes; arrow, endothelial cell; green arrowhead, astrocyte. Scale bars: 25 μm.

The ON is intimately surrounded by the developing leptomeninges (arachnoid mater and pia mater) consisting of two or three layers of fibroblasts, tightly packed, with thin but rather dense collagen fibers (Figure 2A and Figure 3A,B). Pia mater is represented by a denser layer in intimate contact with the nerve, whereas the arachnoid has two or three layers of fibroblasts loosely packed situated at the periphery. Collagen fibers are few compared to the adult organ, but the cellular population is abundant in this gestational age. Those cells will produce the collagen later on. At this moment the pachymeninx (dura mater) is just a thick condensation (3–4 times thicker than the leptomeninges) of the neighboring mesenchyme. The structure suggests fibroblastic differentiation and displays a circular disposition around the ON. Note the absence of the subarachnoid and subdural spaces.

Inside the leptomeninges, one can observe blood vessels indicating the leptomeningeal blood vessels which have a capillary aspect. They can be observed on the entire length of the nerve (Figure 3B).

#### 3.1.2. The Internal Structure

Inside the nerve, in tight contact with the nervous cells and the glia, one can observe the CRA which runs along the nerve, providing metabolic support for both the nerve but also for the retina. This main artery gives numerous collaterals that also run toward the retina. On a close examination with 100× objective, we noted that the future artery is now only a continuous type of capillary, as follows: the endothelial cells make up the walls of the vessel positioned on a basement membrane (Figure 3C).

**Figure 4:**
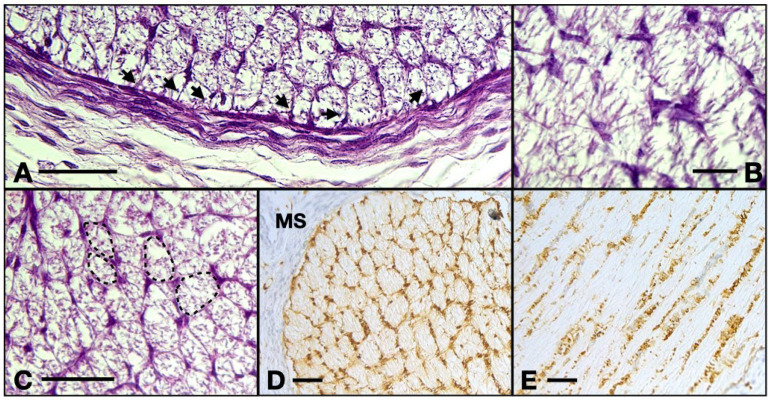
(**A**) 13 WG, the outermost cells emit processes perpendicular to the epineurium and at equal distances to one another, forming a continuous isolation layer. (**B**) 13 WG, astrocytes with big triangle-shaped cell bodies with granular cytoplasm form the inside of the nerve. (**C**) 13 WG, the honeycomb structure of the tubes in the transverse section. Every tube is formed by the projections which emerge from 4–5 cell bodies. (**D**) frontal section of a 14 WG with S100 staining shows that all the cells and their processes are positive for this protein. (**E**) transverse section of a 14 WG with S100 shows positive cells that form tubes in which bundles of axons reside. MS, orbital mesenchyme; arrows, astrocyte processes. Scale bars: A, C, D, and E = 50 μm; B = 20 μm.

Also, we observed that the majority of cells that form the inside of the ON consist of very ramified glial cells, with their central bodies establishing contacts with one another in a reticular manner. The cell bodies are rather big and have various elongated shapes with acidophilic cytoplasm, sometimes with basophilic granules inside that can also be present in the proximal part of the processes. The processes are voluminous at the base and rapidly become narrow and irregular toward the extremity. We observed that the processes establish a reticular structure within the entire mass of astrocytes (Figure 4).

Table S100. positive cells make up a honey-comb-like pattern. Four or five astrocytes emit processes that contact each other and form tubes that will house the axons, structures which are evident also in the HE-stained sections. In the longitudinal aspect, we identified narrow longitudinal tubes made up of astrocytic bodies aligned with very few transversal processes. There are also a few cells negative to S100 with an astrocytic appearance which could indicate the presence of protoplasmic subtype.

**Figure 5:**
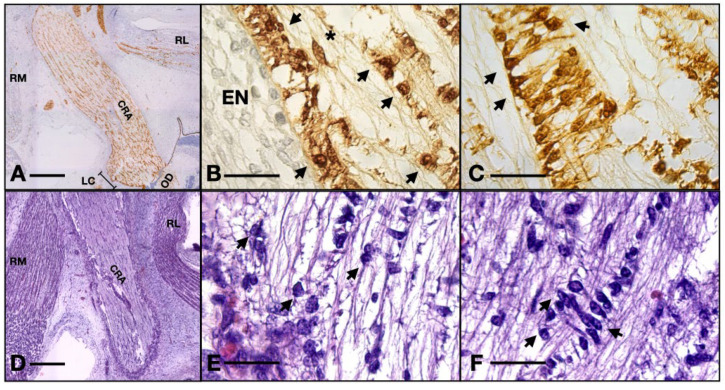
Serial sections of a 14 WG stained with HE versus S100: (**A**,**D**) overview of the orbit; (**B**,**E**) detail of the glia limitans; (**C**,**F**) details of the blood–nerve barrier. Note how the processes wrapping the artery are not evident in the HE stain. RM, rectus medialis; RL, rectus lateralis; CRA, central retinal artery; LC, lamina cribrosa; OD, optic disk; EN, epineurium; arrows, astrocyte nuclei; asterisk, fibrocyte. Scale bar: A and D = 400 μm; B, E, C, and F = 50 μm. One can observe the nuclei of ganglion cells and the flow of axons coming from the axonal layer going toward the optic nerve.

We also note that perivascular cells extend processes that come in tight contact with the endothelial cells, almost completely isolating the vascular elements (Figure 3C). The described structure represents the embryonic aspect of the blood–nerve barrier. In the S100 stained sections, the barrier and its tightly wrapped architecture are even more evident (Figure 5C,F). The glia limitans is now represented by astrocytes lodging in the peripheral region that emit processes that form an almost continuous sheath under the leptomeninges (Figure 5B,E). In the longitudinal aspects, the processes that form the outer sheath of the nerve are parallel to one another and perpendicular to the meninx, equally distanced, coming from cell bodies that form the outermost astrocyte tube at the periphery of the ON. In the transverse aspect, the processes are also perpendicular to the epineurium and tend to have a slightly disorganized pattern (Figure 5A,D). An important observation is that there was a small number of cells that did not stain positive for S100, neither in the cytoplasm nor in the nucleus. These cells are less ramified and have a bigger oval nucleus, characteristics that indicate the presence of fibrous astrocytes. No myelination was seen around the axons of the ganglion cells (that form the ON) and no other glial cell component was evidently observed except the astrocytes that we already described.

**Figure 6:**
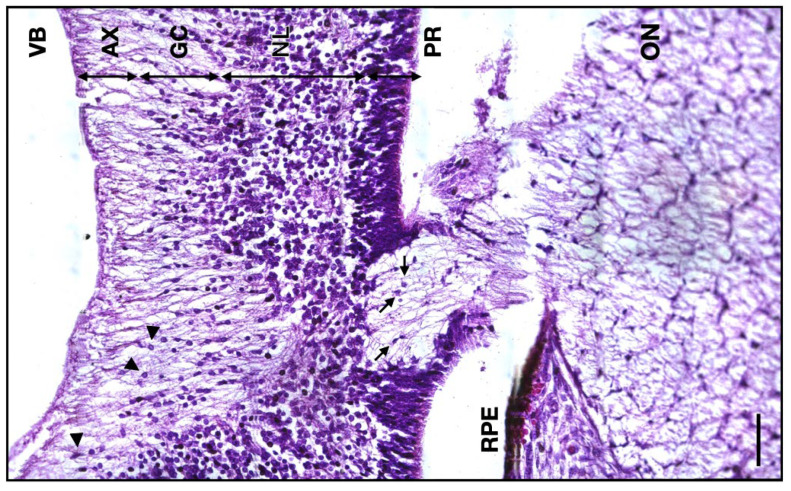
Micrograph of the 14 WG junction between the developing retina and the ON. VB, vitreous body; AX, axon layer; GC, ganglion cells layer; NL, neuroblastic layer; PR, photoreceptor layer; RPE, retinal pigmented epithelium; ON, optic nerve; arrowheads, ganglion cells; arrows, astrocytes. Scale bar: 50 μm.

The thickness of the 13 WG axonal retinal layer has a median value of 64 μm (IQR 7.25; *n* = 6), whereas for the 14 WG, the thickness median value was 39 μm (IQR 7; *n* = 5). At the junction between the ON and the retinal ganglion layer, the glial cells are less present. There are also no photoreceptors, the retina being formed by only a thick neuroblastic layer, the scarce ganglion cell layer, and the axonal layer toward the interior (Figure 6).

### 3.2. 10 WG Group

#### 3.2.1. Surrounding Tissue and Elements

**Figure 7:**
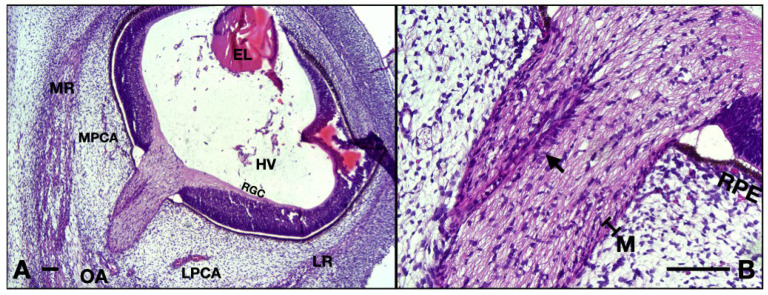
10 WG micrographs: (**A**) overview aspect of the eye and optic nerve; (**B**) detail of the ON and CRA. EL, eye lens; MR, medial rectus; LR, lateral rectus; OA, ophthalmic artery; MPCA, medial posterior ciliary artery; LPCA, lateral posterior ciliary artery; HV, hyaloid vasculature; RGC, retinal ganglion cell layer; RPE, retinal pigmented epithelium; M, meninges; arrow, central retinal artery. Scale bar: 100 μm.

The transverse width of the ON has a median value of 273.5 μm (IQR 8.25; *n* = 10). At this age, the hyaloid vessels are still present in the vitreous. The vessels around the ON are visibly smaller with thicker walls (partially due to the rather early aspect of the endothelium with taller cells with round heterochromatic nuclei). Very small nerves can be identified in the vicinity of the ON. There is no separation between the pachymeninx and leptomeninges, everything is now just a thin primordium of two maximum three layers of fibroblasts in close contact with the ON, lacking the leptomeningeal vessels. Densification of the surrounding mesenchyme as noted in the embryos included in the 14 WG and 13 WG could not be documented (Figure 7A).

#### 3.2.2. Internal Structure

At this age, inside the ON one can observe the CRA, in a more eccentric position. The endothelium has an early aspect presenting taller endothelial cells with round heterochromatic nuclei resulting in a narrower lumen compared to older stages. We cannot note any processes contacting the CRA to form the blood–nerve barrier, but numerous glial nuclei are neighboring the vessel. The internal glia appear to be less ramified with fewer processes, and they create a not so arranged architecture. The columns formed by the nuclei are not perfectly parallel with one another and the nerve fibers running inside are not that parallel either. Also, the structure lacks the astrocytic processes going perpendicular to the epineurium, namely, the embryonic glia limitans (Figure 7B). At the level of lamina cribrosa, the cellular nuclei appear scarce. The thickness of the axon retinal layer has a median value of 26 μm (IQR 4.5; *n* = 10). All these aspects suggest that the optic nerve is found in a disorganized stage compared to the 14 and 13 WG.

### 3.3. 8 WG Group

#### 3.3.1. Surrounding Tissue and Elements

For this age, the width of the ON measured in the same way as for the other groups had a median value of 255.5 microns (IQR 12, *n* = 9).

**Figure 8:**
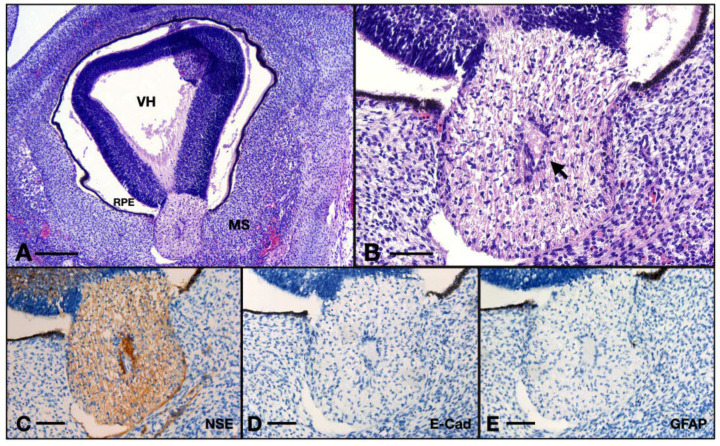
Aspects of the 8 WG ON: (**A**) overview of the eye and orbit; (**B**) detail of the ON junction with the retina from (**A**); (**C**) ON immunohistochemistry with NSE; (**D**) ON immunohistochemistry with E-cadherin; (**E**) ON immunohistochemistry with GFAP. VH, vitreous humor; RPE, retinal pigmented epithelium; MS, intraorbital mesenchyme; arrow, centroretinal artery. Scale bar: A = 250 μm; B–E = 75 μm.

The surrounding mesenchyme here has a blastic appearance with a high density of tachychromatic nuclei. Note that the cells are not ramified at all compared to the embryos of 10 WG. The meninges are almost not differentiated, we only note one thin layer of agglomerated fibroblasts with round and big nuclei, the cells are very tightly packed, and the structure completely lacks the collagenous matrix. No leptomeningeal vessels could be identified. There are no evident vascular or nervous structures inside the orbit (Figure 8A,B).

#### 3.3.2. Internal Structure

The CRA is also present here, but the endothelium is made of round or cubic cells with big and round central nuclei (as opposed to the 14 WG where the endothelium is made up of flat and elongated cells). The blood–nerve barrier in this case is very thick, many cells agglomerate at the border of the vessel but no evident processes contact the endothelium. The reticular architecture that was evident in the 14 WG is completely absent here. The internal cells have very few processes almost only in the transverse direction, they do not contact each other and they exhibit round heterochromatic nuclei, indicating a small grade of differentiation. The outermost layer of astrocytes does not have processes to form the glia limitans, but we do observe an agglomeration of cells at the periphery of the ON. The thickness of the axon retinal layer spans a median value of 50.5 μm (IQR 6.5; *n* = 9).

The ON was positive for NSE. One could observe the thin axons inside, going within the matrix built by the glia. The ON at this age was negative for GFAP and also for E-cadherin, whereas the positive controls were also negative for GFAP but positive for E-cadherin (Figure 8C–E).

## 4. Discussion

The morphogenesis of the ON in the atmosphere of orbital soft tissue is challenging to elucidate, as the organ develops relationships with surrounding structures, depending on the gestational age it is spotted on. Therefore, our attempt to describe changes in an antichronological way, thus, succeeds in explaining each and every change, as well as the motivation for which they occur at a certain moment.

The second half of the first-trimester embryology of the ON surpasses the organogenesis period for most of the structures in question, many of them, inside and outside the ON, being already formed. During the entire studied period, the ON goes through both a maturation and differentiation process happening in tandem.

Our findings indicate that the ON is gradually increasing in size from the 8th week until the 13th week, almost tripling in size. This is consistent with the increase in thickness of the RGC axonal layer. In the 14 WG, however, there will be a loss in diameter (from 640 μm to 450 μm, so a loss of 200 μm). Similarly, this happens for the RGC axonal layer (from 63 μm to 40 μm, so a shrinkage of approximately 20 μm), both structures becoming smaller by a factor of one-third. This is in accordance with the conclusions of Provis et al. [9]. As with other specialists who studied this process, we were not able to determine whether this shrinkage is the result of axon apoptosis or of glial cell apoptosis, which could be needed for creating more space for axon migration [7,9,18].

The meninges undergo a maturation process, as follows: from just an agglomeration of one-layered fibroblasts to double-layered meninges with intrinsic vessels in the 14th week. These capillary vessels run along the nerve to ensure that blood provides the metabolic substrates for fibroblast’s collagen secretion and their differentiation. We suggest that the factor inducing the split between the pachymeninx and leptomeninges and also that induces the collagen matrix secretion is the appearance of the intrinsic blood vessels. Interestingly Dasgupta and Jeong claim that by 6 WG the meninges covering the brain are organized as three distinct layers, whereas the ON has a one-layered meninx up to 10 weeks [13]. The delay could be attributed to molecular factors, and it is yet to be discussed. This rather divergent conclusion is unified by Couly and Le Douarin, who experimentally demonstrate that the meninges covering the forebrain (optic nerve included) originate in the diencephalic neural crest and extend forwards [19]. So, a delay in development based on the position can be reasonable.

As early as 8 WG, the axons inside the ON exhibit adult and differentiated immunohistochemical characteristics, being positive for NSE [20]. During the fetal brain development, the main enzyme first noted is NNE. Later on, the main enzyme will be switched to NSE, suggesting that the nervous cell is now a differentiated mature cell [21]. Also, the axons are negative to E-cadherin (a marker of pluripotent and undifferentiated neurons) further supporting the adult nature of the axons of RGC [22,23].

Concerning the astrocytes, we used GFAP to highlight the presence of mature astrocytes [24]. Interestingly, the ON was not at all positive, also the positive control (brain fragment of 8 WG) was negative as well. To test the antibody, we also used adult brain specimens which certified that the antibody worked properly. Therefore, we acknowledge that the astrocytes of the 8 WG embryo group do not express the GFAP, which comes into agreement with Guo et al., who claim the absence of the protein in the early developing spinal cord until the 9–10 WG [25].

Contributing to the immunohistochemical study is the antibody against S100 which is a molecule implicated in the migration and differentiation of astrocytes and is also expressed in two populations of glial cells in the developing central nervous system [26,27,28,29]. Indeed, we could identify a small number of cells, bearing a morphology resembling the other astrocytes but negative to S100. This could indicate the presence of protoplasmic astrocytes.

The glial architecture evolves from a disorganized state at 8 WG becoming increasingly intricate at 13–14 WG when the astrocytic tubes are fully formed. We already stated that at 8 WG axons are already adult, whereas astrocytes are not differentiated and morphologically immature, a conclusion in accordance with Raff and Miller, who proved that astroglia need axons to develop and mature [30]. On the other hand, Silver and Robb demonstrated in animal models that axons need the astrocytic tubes for normal migration toward the optic chiasm and further development under normal conditions [31]. We postulate that although astroglia are present at 8 WG under its simple, disorganized, and blastic form, and they need adult axons to provide signals for their further development into ramified cells that form the intricate tubes.

The formation of blood blood–brain barrier (extrapolating to the blood–nerve barrier) in mice is divided by Haddad et al. into the following three phases: angiogenesis, differentiation, and maturation [32]. Out of these, only the last two could be visualized in our time window. During the angiogenic phase (3–5 WG), neural progenitors secrete vast quantities of Vascular Endothelial Growth Factor signaling the migration of endothelial cells that form immature vessels [33,34]. The next step is the differentiation period which begins at 7 WG. Under the action of endothelial factors, astrocytes and pericytes are attracted to the vessel, astrocytes begin to express foot processes that will gradually isolate the artery under the pericyte’s influence [32,35]. As we found at 8 WG there are no processes contacting the CRA but there is a tight agglomeration of nuclei in the perivascular region. This aspect suggests the unspecialized nature of the early glial cells, not being yet able to form the morphological blood–nerve barrier. After this period, the barrier is morphologically complete, but maturation is expected, as many modifications concerning the functionality of the barrier should take place, making it completely functional by the third trimester [32]. This process is marked by an increasing number and redistribution of tight junctions stabilizing the neurovascular unit, programmed by the crosstalk between astrocytes, endothelial cells, neurons, and microglia [32,36].

## 5. Conclusions

The embryologic development of the ON in the window between 8 WG and 14 WG consists of a histological and cytological maturation process. All the structures in the adult nerve do have an early homolog, as early as the 8th week. First, the ON appears as a small, thin structure with a fragile meningeal primordium made of only one fibroblastic layer, and inside it exhibits a thick-walled early artery. Also, at this age, the immunohistochemical analysis proves that the axons coming from the RGC layer are mature but the glial cells do not express the definitive astrocyte-specific marker, observations in agreement with the blastic, simple, and nonspecialized aspect of the glial cells. In the 10 WG, the glial cells inside begin to emit short and disorganized processes, the structure becomes larger, and the endothelium of the CRA becomes cubic as the lumen widens. Many axons compose the inside of the ON and the glial cells tend to align into the future tubes. At 13 WG the nerve has thick and collagenous meninges divided as pachymeninx and leptomeninges. The glial cells become very ramified and triangular-shaped and organize themselves to form the astrocytic tubes. The CRA now becomes a capillary with a wide lumen surrounded by flat adult endothelium. At this age, we observe for the first time the astrocytes isolating and contacting the vessel via their processes to form the blood–nerve barrier. The 14 WG does not bring any major changes only that the meninges thicken the glial cells become even more ramified also in the transversal aspect and the nerve shrinks in diameter by a factor of one-third.

## Data Availability

Data are contained within the article.

## Abbreviations

CRA	central retina artery
GFAP	glial fibrillary acidic protein
HE	hematoxylin–eosin
NSE	neuron-specific enolase
ON	optic nerve
OS	optic stalk
S100	S100 calcium-binding protein
WG	week gestational
